# Deep Eutectic Solvents in Capillary Electromigration Techniques—A Review of Recent Advancements

**DOI:** 10.3390/molecules30183674

**Published:** 2025-09-10

**Authors:** Michał Pieckowski, Ilona Olędzka, Tomasz Bączek, Piotr Kowalski

**Affiliations:** 1 Department of Pharmaceutical Chemistry, Medical University of Gdańsk, Hallera 107, 80-416 Gdańsk, Poland; ilona@gumed.edu.pl (I.O.); tomasz.baczek@gumed.edu.pl (T.B.); piotr.kowalski@gumed.edu.pl (P.K.); 2 Department of Nursing and Medical Rescue, Institute of Health Sciences, Pomeranian University in Słupsk, 76-200 Słupsk, Poland

**Keywords:** deep eutectic solvents, capillary electrophoresis, separation sciences, green chemistry, enantioseparation, pseudostationary phase

## Abstract

Deep eutectic solvents (DESs) represent a versatile and sustainable class of solvents, characterized by their low volatility, favorable biodegradability, and the ability to tailor their viscosity, polarity, and hydrogen-bonding capacity through the choice of their individual components. These characteristics have established them as powerful media in various analytical extraction and separation processes. This review presents a critical evaluation of the expanding role of DESs within the field of capillary electromigration techniques, summarizing key advancements from 2019 to mid-2025. We synthesize the current literature to delineate the benefits, persistent challenges, and future prospects of integrating DESs into capillary electrophoresis (CE)-based analytical workflows. Specifically, it systematically documents the following: (i) the diverse types of DESs employed in electrophoretic separations, (ii) proposed mechanisms underlying their influence on chiral compound resolution, and (iii) their utilization as separation media and pseudostationary phases (PSP) in capillary electromigration systems. By critically assessing their advantages and drawbacks, this review aims to provide a comprehensive perspective on the application of DESs in modern capillary electromigration techniques.

## 1. Introduction

Deep eutectic solvents (DESs) were first described by Abbott et al., who succeeded in creating a room temperature liquid (freezing point at 12 °C) from two solid components: choline chloride (ChCl) and urea (U) with melting points of 302 °C and 133 °C, respectively, in a 1:2 molar ratio [1]. This phenomenon is attributed to the formation of hydrogen bonds between the DES components, resulting in a significant depression of the melting point. Structurally, DESs are composed of a hydrogen bond acceptor (HBA) and a hydrogen bond donor (HBD) in a defined stoichiometric ratio. The ease and low cost of preparation, low toxicity, and biodegradability of DESs make them attractive alternatives to traditional organic solvents and ionic liquids. Since 2019 [2], DESs have been introduced into capillary electromigration techniques as additives to the background electrolyte (BGE), improving the separation of chiral compounds such as zopiclone, salbutamol, and amlodipine. Although the use of DESs in capillary electrophoresis (CE) is still in its infancy, recently a growing trend in publications on DESs in capillary electromigration techniques has been observed.

## 2. Methodology

This review is based on a systematic search of the scientific literature conducted to identify relevant studies on the application of DESs in capillary electromigration techniques. The search was performed using the PubMed, Scopus, Web of Science, and Google Scholar databases, covering publications from 2019 up to July 2025. A representative search query, combining keywords with Boolean operators, was as follows: (“*deep eutectic solvents*” *OR* “*DES*”) *AND* (“*capillary electrophoresis*” *OR* “*capillary electromigration techniques*” *OR* “*capillary electrokinetic chromatography*”). The screening of retrieved articles was guided by specific inclusion and exclusion criteria. To be included, articles had to be original research papers published in English in peer-reviewed journals, focusing specifically on the application or development of DESs in capillary electromigration. Conversely, review articles, book chapters, conference proceedings, and patents were excluded. Furthermore, studies not directly related to the core topic or those lacking sufficient experimental data or significant theoretical contributions to analytical applications were also omitted to ensure the quality and relevance of the cited literature.

## 3. DESs Preparation

The typical preparation of a DES involves the combination of HBA and HBD in the appropriate molar ratio, followed by mixing and heating the mixture in a glass vessel using a magnetic stirrer or rotary evaporator at 80 °C in a water bath for approximately 2 h, or until a clear, transparent solution is obtained [3]. For DESs composed of ChCl and clindamycin phosphate (CP), five drops of water were added to the mixture prior to heating to break intramolecular bonds in CP, and the heating process was extended to 6 h [4]. In the study by Ding et al. [5], a DES consisting of proline (Pro) and U was prepared with the addition of water to regulate the density of the resulting DES (Pro-U30, containing 30% *m*/*m* water). In the case of DESs composed of cyclodextrins (CD) and malic acid (MA), a drop of methanol was required to break strong hydrogen bonds in the CD and MA molecules and initiate the reaction [6]. Meng et al. [7] prepared a mixture of betaine (B) and urea (U) with water, using a molar ratio of 1:2:2. After the formation of the DES (B-U), water was added in quantity ranging from 20 to 40% (*m*/*m*) to modify the density, followed by vortexing and ultrasonic treatment. Table 1 below presents all up-to-date DESs used in capillary electromigration techniques.

Interestingly, Liu et al. [20] conducted research on the effect of the HBA:HBD ratio (CDs to LA) on the enantioseparation of cations (alkaline drugs), varying the ratio between 1:2 and 1:10. The study investigated the impact of DES on migration time, selectivity factor (α), and resolution of analytes using a BGE containing 7.5% DES, 30 mM phosphate buffer, at pH 3.15. The following conclusions were drawn: (i) as the concentration of HBD increased, the migration times of the analytes decreased, which was associated with an increase in electroosmotic flow (EOF); (ii) an increase in HBA concentration improved the enantioseparation of analytes while simultaneously reducing retention time.

Moreover, Ma et al. [23] investigated the influence of the ratio of TBACl to LbA on the separation of amino alcohol drugs, selecting three ratios (1:1, 1:2, 1:3) for further examination. When the concentration of HBA was low (1:3), the modification of EOF and the improvement in separation were insufficient. In contrast, a high HBA concentration (1:1) resulted in excessively high ionic strength, which affected chiral recognition interactions. Accordingly, a 1:2 ratio was considered optimal for TBACl to LbA. These observations suggest that selecting the optimal stoichiometric ratio of DES components is crucial for manipulating EOF and, consequently, for effective electrophoretic separations.

## 4. Characterization of DESs in Capillary Electromigration Techniques

### 4.1. Spectroscopic Techniques

Fourier-transform infrared (FT-IR) spectroscopy is employed to characterize newly synthesized DESs, with spectra recorded for the DES as well as for its individual components, i.e., HBA and HBD. FT-IR spectroscopy provides insight into the molecular structure and intermolecular interactions within the system. This technique enables the identification of characteristic vibrational bands corresponding to specific chemical bonds. The analysis primarily focuses on shifts observed in the stretching vibrations of hydroxyl (ν(O-H)), amine (ν(N-H)), and carbonyl (ν(C=O)) groups, which serve as direct evidence for the formation of hydrogen bonds. Changes in the position and intensity of these bands in the FT-IR spectra are considered definitive evidence for the formation of the eutectic system [2,3].

Proton nuclear magnetic resonance (^1^H NMR) spectroscopy is also utilized in the structural analysis of DESs, providing information about the chemical environment of protons in both HBA and HBD molecules. ^1^H NMR allows for the assessment of changes in chemical shifts (δ) and signal integrals, enabling the monitoring of hydrogen bond formation and dynamic proton exchange processes. Furthermore, ^1^H NMR spectra can be used to determine the stoichiometry of the DES and to verify the purity and stability of the eutectic system [4].

Complementing these techniques, carbon-13 nuclear magnetic resonance (^13^C NMR) spectroscopy is a valuable tool for DES characterization. It enables a detailed analysis of the chemical environment of carbon atoms within the HBA and HBD molecules. By identifying changes in the chemical shifts of carbon atoms, particularly those in carbonyl, hydroxyl-adjacent, and heterocyclic groups, it is possible to infer the participation of these centers in the hydrogen-bonding network of the DES. ^13^C NMR is particularly useful for assessing the stability of the DES and for detecting any potential chemical reactions between its components [5].

In summary, FT-IR is most effective at elucidating the changes in hydrogen bonding between HBA and HBD. ^1^H NMR allows for the quantitative and qualitative assessment of proton behavior in the DES, whereas ^13^C NMR complements the ^1^H NMR data by confirming intermolecular interactions that may not be apparent in the proton spectra.

### 4.2. Classification of DESs Used in Capillary Electromigration Techniques

**Figure 1:**
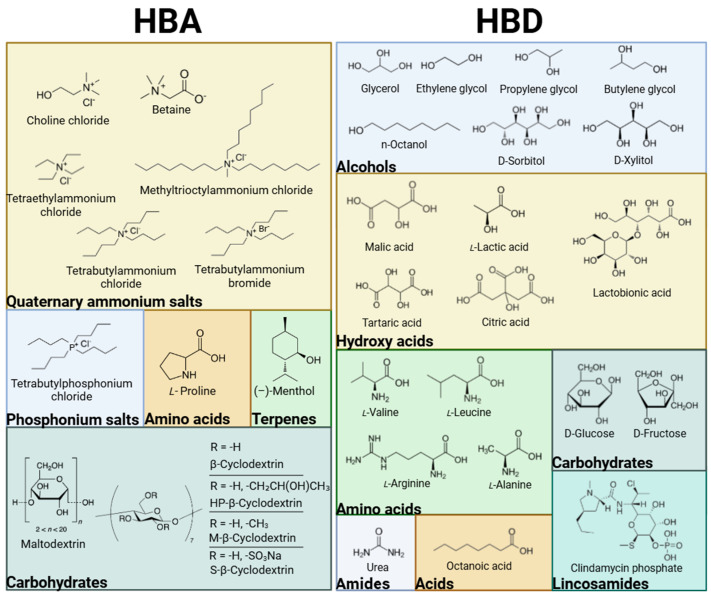
Classification of HBA and HBD components for DESs employed as additives in capillary electromigration techniques.

The rapidly growing interest in DESs and their application in CE has led to the introduction of a diverse range of HBAs and HBDs. These components vary in their molecular structure and origin, being either naturally derived or fully synthetic. Figure 1 below presents a classification of the DESs utilized in capillary electromigration techniques.

## 5. Enantiomeric Separation Using DESs as Additives

The first application of DESs as additives for chiral separations was reported in 2019. In their work, Y. Mu et al. introduced DESs based on ChCl combined with either U or various diols into the BGE. By employing a binary system composed of DES and β-CD—a technique referred to as DES/CD-modified capillary zone electrophoresis (DES/CD-CZE)—they successfully resolved racemic mixtures of zopiclone, salbutamol, and amlodipine. Notably, the addition of a ChCl-U DES to the BGE resulted in a nearly four-fold enhancement in the resolution (*Rs*) for racemic zopiclone. The resolution was calculated using the standard equation: *Rs* = (*t*_2_ − *t*_1_)/0.5(*w*_2_ + *w*_1_), where *t* represents the migration time and *w* is the peak width at the base. The authors proposed two possible explanations for the synergistic effect of the binary DES/β-CD system: (i) alterations in the ionic strength of the BGE upon DES addition, and (ii) a dynamic coating of the inner capillary wall by the DES. These phenomena were suggested to cause the suppression or even reversal of the electroosmotic flow (EOF) [2].

To elucidate the mechanism by which DESs enhance chiral separations, S. Deng et al. investigated the interaction between the analyte propranolol and the chiral selector carboxymethyl-β-cyclodextrin (CM-β-CD) using fluorescence spectrophotometry. The study compared the system in the absence and presence of a ChCl-LA DES. Initial observations confirmed that CM-β-CD quenches the fluorescence of propranolol, and this quenching effect was significantly more pronounced upon the addition of DES. By applying the Benesi–Hildebrand method to the fluorescence data, the binding constant (K) for the PPL-CD inclusion complex was calculated. The results revealed a substantial increase in binding affinity: the constant rose from K = 23 M^−1^ without the DES to K = 142 M^−1^ with 5% ChCl-LA. The authors attribute this significant increase in the binding constant to a “squeezing effect,” where DES promotes the inclusion of propranolol molecules into the CD cavity. This enhanced formation of the host–guest complex strengthens the chiral recognition process, thereby leading to improved enantiomeric resolution [3]. CM-β-CD (carboxymethyl-β-CD) possesses a negative charge in slightly acidic media (pH = 4.0) due to deprotonation of carboxymethyl groups; therefore, the technique should be classified as DES modified cyclodextrin electrokinetic chromatography (DES-CDEKC).

In contrast, Zhu et al. [6] and Liu et al. [20] investigated SUPRADESs formed from non-ionic CDs (β-CD, methyl-β-CD, hydroxypropyl-β-CD) and organic acids such as L-lactic acid. In these studies, the SUPRADESs act as neutral chiral selectors, and the separation mechanism corresponds to the SUPRADES-CZE mode. Here, the non-ionic CDs do not contribute to the electroosmotic flow or act as a pseudostationary phase; instead, chiral discrimination is achieved primarily through enhanced supramolecular interactions—such as hydrogen bonding and hydrophobic effects—between the analytes and the SUPRADES components. Both Zhu et al. and Liu et al. demonstrated that the formation of SUPRADESs significantly increases enantioselectivity compared to the use of CDs or DESs alone, as confirmed by higher binding constants and greater differences in binding free energies (ΔΔG) between enantiomers. Molecular modeling and spectroscopic analyses further revealed that the enhanced chiral recognition in SUPRADES systems is due to amplified non-covalent interactions and increased host–guest encapsulation variance.

In summary, the key mechanistic difference lies in the charge and role of the CD: in SUPRADES-CDEKC [17,18], the anionic S-β-CD acts as a mobile, charged pseudostationary phase, enabling both electrophoretic and chiral interactions. In contrast, in SUPRADES-CZE [6,20], non-ionic CDs serve as neutral chiral selectors, with separation governed by supramolecular host–guest chemistry.

Another novel approach utilizes DESs not as the primary chiral selector, but as additives to a conventional chiral selector in the BGE. This methodology, which can be termed DES-assisted chiral selector-CZE (DES/CS-CZE), is fundamentally different from systems where SUPRADES acts as the sole selector. Seminal works by Zhang et al. [22], using maltodextrin as the CS, and Ma et al. [25], employing the antibiotic CP, exemplify this strategy.

The underlying mechanism in the DES/CS-CZE mode is not based on creating a new primary selector, but on modulating and enhancing the chiral recognition capabilities of the existing one. Both studies conclude that the presence of DES significantly improves enantioseparation compared to the system with the chiral selector alone. The proposed mechanism involves the DES modifying the solvation microenvironment, leading to the formation of more stable and structurally compact ternary complexes involving the analyte, the primary chiral selector, and the DES components. As Ma et al. theorize, DESs “promote the combination” of the analyte and the selector. Furthermore, when the DES itself is composed of chiral precursors (e.g., L-lactic acid, L-valine), it can provide secondary chiral interactions, further amplifying the enantioselectivity. In this capacity, the DES functions as a high-performance “co-selector” or a “performance-enhancing additive” that refines the primary chiral recognition event, rather than initiating it.

In a study by Ma and Zhang [4], CP was, for the first time, directly utilized as a component of a DES serving as a chiral selector in capillary electromigration techniques. Specifically, CP acted as the HBD in a DES system with ChCl as the HBA, and this novel DES was applied in nonaqueous capillary electrophoresis (DES-NACE) to enhance the enantioseparation of twelve amino alcohol drugs. The incorporation of CP into the DES resulted in significantly improved resolution and peak shapes compared to the use of unmodified CP, enabling complete baseline separation for the majority of analytes. Systematic optimization of the NACE conditions yielded an optimal buffer consisting of methanol containing 65 mM ChCl-CP DES, 45 mM Tris as the BGE, 0.3 M NaOH, and 10% (*v*/*v*) water, which provided a balance between high enantioselectivity and analysis times of less than 25 min at an applied voltage of 24 kV. This approach demonstrates the potential of using chiral selectors (other than CDs) as integral components of DESs to further enhance chiral separation performance in CE.

A further evolution in the use of DESs as performance-enhancing additives involves their integration with nanomaterials to create novel functional platforms. This is exemplified in the work by Wang et al. [27], who introduced multi-walled carbon nanotubes modified with DES (DESs@CNTs) as buffer additives. In their system, which employed maltodextrin (MD) as the primary chiral selector, the mechanism extends beyond simple microenvironment modulation. The hydrophilic DESs serve to functionalize the hydrophobic nanotube surface, ensuring stable dispersion and creating a high-surface-area scaffold that adsorbs and enriches the primary selector. This synergistic approach not only resulted in a significant improvement in enantiomeric resolution but also allowed for a substantial reduction in the required concentration of the primary selector, achieving optimal separation with just 3% (*m*/*v*) MD, compared to the 4–6% (*m*/*v*) typically required in conventional systems. This enhancement was achieved using a low concentration of the functionalized additive, with 0.5% (*m*/*v*) DESs@CNTs identified as optimal under acidic conditions (pH 3.0). Furthermore, the system demonstrated high stability, with intra-day and inter-day relative standard deviations (RSDs) for resolution values below 5.3% and 6.8%, respectively. This strategy represents a shift from employing DESs as simple, soluble co-selectors to utilizing them as key components in the design of hybrid, solid-phase-assisted chiral recognition systems.

Table 2 provides a summary of all studies in which DESs are employed as additives to the BGE, functioning either as the primary chiral selector or as agents that improve selector–analyte interactions.

## 6. DES as Separation Medium in CZE and CEKC

There is a growing interest in using DESs as the main medium in the BGE instead of water. However, defining DES as an alternative pseudostationary phase (PSP) is problematic. By definition, a PSP in capillary electromigration techniques must possess several key attributes. First, it must exhibit an intrinsic electrophoretic mobility (μ_ep (PSP)_) distinct from that of the electroosmotic flow (μ_eof_), as this velocity differential is fundamental to the separation mechanism. Furthermore, a PSP must be electrically charged. This requirement is well exemplified by the mechanistic contrast between CD-EKC, where charged cyclodextrins form a bona fide PSP, and CD-CZE, where neutral cyclodextrins migrate passively with the EOF. Moreover, separation in EKC relies on the differential partitioning of analytes between the BGE and the PSP. An analyte with a stronger affinity for the PSP (i.e., a higher partition coefficient, K) resides within it for a longer duration, causing its net migration velocity to approach that of the PSP. This dynamic establishes a finite migration window between the EOF (t_0_, representing non-interacting species) and the PSP (t_mc_, representing fully associated species), within which all partitioning analytes must elute. Finally, a PSP must be uniformly dissolved or dispersed throughout the BGE to form a macroscopically homogeneous mixture, ensuring reproducible separation conditions along the entire capillary length.

The development of DESs as PSPs (or separation media) in capillary electromigration techniques can be divided into three main directions: (i) DESs based on mixtures of amino acids and urea [5,7,24], (ii) hydrophobic DESs (HDESs) [14,15,19], and (iii) HDESs as the oil phase component in microemulsions (ME) [21].

In the first case, based on the established criteria for a PSP, the proline–urea-based DES (Pro-U30) described by Ding et al. [5] functions not as a PSP, but as a novel primary separation medium that replaces conventional aqueous or organic solvents. In the initial part of their study, Pro-U30 serves as the bulk solvent for CZE. A true PSP must be a charged entity with its own electrophoretic mobility, distinct from the EOF. However, the Pro-U30 medium, composed of neutral and zwitterionic components, lacks the intrinsic net charge required to act as such; the authors report that Pro-U30, with a viscosity of 2–8 mPa·s and a high dielectric constant of 254.6, supports a measurable electroosmotic flow (μ_eof_ = 0.55 × 10^−4^ cm^2^/V·s), and the migration order of analytes (v(cations) > v(EOF) = v(neutrals) > v(anions)) matches that of standard aqueous CZE, relying solely on the analytes’ charge-to-size ratio. This is critically evidenced by the authors’ inability to resolve structurally similar isomers, confirming the absence of the partitioning mechanism that defines a PSP. The subsequent transition to MEKC further clarifies this distinction. It is the addition of the surfactant SDS to the Pro-U30 medium, not the DES itself, that introduces a true PSP in the form of charged SDS micelles. These micelles provide the necessary partitioning environment to resolve the previously inseparable isomers. Thus, the separation mechanism in this work is best described as CZE in a DES-based medium, rather than as a PSP- or MEKC-type system (DES-based CZE or DES-based MEKC).

The first application of a DES as a PSP can be attributed to Li et al. [15], who employed a HDES composed wth the mixture menthol and octanoic acid (Men:OA). This work effectively established a novel EKC mode. Firstly, the system meets the requirement of homogeneous dispersion. The authors demonstrate that the water-immiscible Men:OA HDES can be dispersed into a stable aqueous buffer via ultrasonication, remaining stable for at least 48 h. The formation of colloidal micro-droplets was visually confirmed by the Tyndall effect, and their size was precisely quantified by dynamic light scattering (DLS) and transmission electron microscopy (TEM), revealing a mean diameter of 368 nm and a range of 300–500 nm, respectively. Secondly, the HDES introduces an effective partitioning mechanism. This is evidenced by its ability to resolve structurally similar aromatic acids, including isomers that were inseparable in the CZE mode. Moreover, in a “dual-PSP” system with HP-γ-cyclodextrin, the addition of the HDES drastically improved separation, as illustrated by specific resolution (Rs) values. For instance, the Rs for the isomer pair 2-naphthaleneacetic acid (2-NAC) and 1-naphthaleneacetic acid (1-NAC) increased from 2.45 to 3.98, while for 2,7-naphthalenedisulfonic acid (2,7-NDS) and 1,5-naphthalenedisulfonic acid (1,5-NDS), it increased from 4.49 to 8.66. This unambiguously demonstrates the unique contribution of the HDES to the system’s selectivity. The tunability of the system was also shown, as varying the HDES concentration from 0.1 to 0.5% allowed for control over selectivity and analysis time. Optimal conditions (0.3% Men:OctA, pH 10, 10 kV) enabled the baseline separation of 13 similar aromatic acids in under 30 min with high efficiency (100,000–150,000 theoretical plates). Thirdly, the most controversial aspect is the authors’ explanation of the migration order and the influence of the HDES on the analytes’ electrophoretic mobility. By establishing a velocity hierarchy where v(HDES) = v(EOF) > v(anionic analyte), they premise that the HDES is the fastest-moving entity towards the detector. Based on this logic, they correctly state that a stronger interaction with the HDES should lead to faster migration. However, they then use this premise to explain their observation that “the presence of the HDES in the running buffer decreased the effective electrophoretic mobility (μ_e_ff),” which is a fundamental logical contradiction. A general decrease in the mobility of all species could be partially attributed to global effects like increased BGE viscosity or dynamic capillary coating, which suppress the EOF, analogous to what occurs in non-ionic MEKC with Brij-35 micelles. However, such effects cannot explain the differential decrease in mobility that leads to enhanced separation. Alternatively, another mechanism can be suggested: at pH 10.0, both the analytes and the HDES droplets (due to the ionized octanoic acid, p*K*_a_ ≈ 4.9) are negatively charged and have their own electrophoretic mobility towards the anode (opposite to the EOF). The HDES phase is a “slower-moving phase” than the analytes (or moves at a similarly low velocity). When analytes interact with this slow phase, their average velocity towards the detector further decreases. However, this hypothesis requires experimental confirmation.

In a follow-up study, Li et al. [15] demonstrated the successful application of the (−)-Men:OA HDES as a neutral PSP for chiral separations in an acidic medium (pH 2.0–3.0). Under these conditions, the HDES forms a stable dispersion of neutral micro-droplets with an average diameter of 363 nm. The HDES functions as a powerful modulator in a dual-PSP system with an anionic chiral selector, CM-β-CD (CD/HDES-EKC mode). The addition of just 0.3% (*m*/*v*) HDES to the background electrolyte enabled the baseline enantioseparation of six chiral drugs that were previously unresolved with CM-β-CD alone. This demonstrates that the HDES introduces a crucial, non-chiral partitioning mechanism, primarily driven by hydrophobic interactions. Crucially, the authors’ mechanistic description aligns with their experimental observations. They correctly identify the HDES as a neutral PSP migrating with the EOF and note that its presence significantly accelerates the migration of the positively charged analytes (or their complexes with the slower-moving, anionic CM-β-CD). This acceleration is a direct consequence of the analytes partitioning into the fast-moving, neutral HDES phase. Optimal conditions, using 2–4% CM-β-CD and 0.3% HDES at pH 2.5 and 20 kV, yielded successful chiral separations. This work validates the use of HDES as a “switchable” PSP, whose charge and migration behavior can be controlled simply by adjusting the BGE pH, thereby providing a versatile tool for designing advanced EKC systems.

In a subsequent study, Xu et al. [19] introduced a second, fundamentally different type of HDES, composed of methyltrioctylammonium chloride and octanoic acid (N_8881_Cl:OA), as a PSP. This HDES also meets the core criteria for a PSP, forming a stable dispersion of micro-droplets with an average diameter of 386 nm and introducing a new partitioning mechanism that significantly improved the enantioseparation of four model drugs when used in a dual-PSP system with CM-β-CD. The optimal HDES concentration was found to be 0.15% (*m*/*v*). The critical distinction of this work lies in the nature of the PSP’s charge. Despite the authors describing the phase as “electrically neutral,” the chemical reality of the system, operating at pH 2.5, dictates otherwise. The N_8881_Cl component is a quaternary ammonium salt, possessing a permanent cationic charge regardless of pH, while the OA is neutral. Consequently, the HDES is, in fact, a cationic PSP. This cationic nature correctly explains the experimental results. The authors observe a significant acceleration of the (protonated) analytes’ migration upon addition of the HDES. A cationic PSP possesses its own intrinsic electrophoretic mobility in the same direction as the EOF, creating a “fast-moving” phase. Interaction with this phase logically leads to the observed acceleration.

Jin et al. [21] demonstrated an advanced application of a HDES by incorporating it as the oil phase within a microemulsion, thereby establishing a novel DES-in-water microemulsion electrokinetic chromatography (DES/W MEEKC) method. In this system, the PSP is not the HDES itself, but a complex, multi-component microemulsion droplet. The optimized PSP consisted of 0.5% HDES (DL-menthol:*n*-octanol, 1:1 molar ratio) as the oil core, stabilized by 3.3% of the anionic surfactant SDS and 6.6% 1-butanol as a co-surfactant in a 15 mM borate buffer (pH 8.5). The negative charge imparted by the adsorbed SDS molecules rendered the microemulsion droplets an anionic PSP, which migrates with its own electrophoretic mobility against the EOF.

The authors correctly identified and validated the MEEKC mechanism by observing the classic migration order: EOF, analytes, microemulsion droplets. Crucially, they quantified the separation window, obtaining a value of 5.7, which confirms the existence of a robust electrokinetic chromatography system. This innovative method enabled the baseline separation of nine phenolic compounds in under 18 min. This study exemplifies a sophisticated use of HDES, not as a standalone PSP, but as a tunable, green core component within a well-defined MEEKC framework.

An overview of DESs employed as separation media in capillary electromigration techniques is provided in Table 3.

## 7. Selected Aspects Related to Capillary Electromigration Techniques Based on DESs

### 7.1. Advantages of DESs

Sustainable development in analytical chemistry, including the minimization of the environmental impact of chemical processes, has become one of the key paradigms in the design of separation methods. In this context, DESs are gaining increasing significance as an alternative to traditional organic solvents commonly used in electrophoretic techniques. DESs belong to the group of “green solvents”—they are easy to synthesize, non-toxic, biodegradable, and typically inexpensive. They are usually prepared by simply mixing two or more components.

Importantly, the synthesis of DESs does not require the use of solvents, catalysts, mechanical energy, or additional purification steps, making the process environmentally friendly and energy efficient. Their application allows for the reduction of organic solvent consumption, such as methanol or acetonitrile, aligning with the principles of green analytical chemistry [5,24]. Ding et al. employed DESs as a sustainable alternative to toxic organic solvents in CE. Particular attention was given to a Pro-30U, which demonstrated not only a high capacity for dissolving β-CD (up to 30% *m*/*v*) but also electrophoretic selectivity comparable to or exceeding that of traditional aqueous systems. This DES, composed of natural and biodegradable components (an amino acid and a nitrogen fertilizer), enabled the elimination of aprotic organic solvents such as DMF, DMSO, or FA. Notably, the Pro-U-based system exhibited reduced UV baseline disturbances and greater compatibility with β-CD, without the adverse effects on pseudo-stationary phase interactions commonly encountered with organic solvents. These studies provide compelling evidence that DESs can function as primary separation media, representing a significant step toward more ecological and safer analytical chemistry.

Another important advantage confirming the “green” character of DESs is their low toxicity and high biodegradability. In particular, mixtures based on biocompatible components, such as choline (a B-group vitamin metabolite) and glycerol (a by-product of biodiesel production) [3,8,9,10,11,12], are considered safe for both the user and the natural environment. Unlike conventional organic solvents (e.g., acetonitrile, DMSO, MeOH), which are volatile, toxic, and often difficult to dispose of, DESs are almost entirely labile under biological conditions and readily degrade in aqueous environments. The incorporation of DESs into the separation buffer enables modification of its physicochemical properties, including polarity, ionic strength, viscosity, and pH. DESs can interact with analytes through hydrogen bonding, van der Waals forces, or complexation, which in turn affects their electrophoretic mobility and separation selectivity.

The tunability of DESs can also contribute to a reduction in the overall consumption of other chemical reagents. They can effectively replace alcohols and aprotic solvents in roles such as stabilizers, complexing agents, or polarity modifiers, while simultaneously enhancing enantioselectivity and improving the solubility of poorly water-soluble analytes, such as antifungal agents or selective serotonin reuptake inhibitors [16,26]. The unique solvating environment of DESs can also enhance the stability of chiral selectors or analytes in solution, minimizing chemical degradation. As demonstrated by Li et al. [15], the use of HDESs, such as (−)-Men:OA, create a PSP for analytes with low water solubility. Furthermore, DESs exhibit inherent buffering properties, which depend on the composition of their components. For instance, mixtures containing lactic acid, urea, or amino acids can influence the acid–base balance of the system. Hu et al. [26] observed that the inclusion of DESs can stabilize pH within acidic or slightly basic ranges, which is often advantageous for maintaining the activity of chiral selectors.

One of the most significant advantages of employing DESs is their potential to enhance detection sensitivity. In UV detection, signal enhancement can result from several mechanisms: increased analyte solubility, shifts in electronic distribution that elevate molar absorptivity, and reduced band broadening due to analyte dehydration. The capabilities of signal amplification have been widely demonstrated. Ioannou et al. [11,12,17] observed that both standard DESs and novel SUPRADES systems led to an increase in detection signal intensity for amphetamine derivatives, attributed to enhanced solubility and stabilization of analyte–selector complexes. Similarly, Jin et al. [21] reported that using a DES as the oil phase in MEEKC enhanced the sensitivity for phenolic acids, with LODs ranging from 0.22 to 1.04 µg/mL. Zhang et al. [22] and Xu et al. [19] also reported that amino acid-based HDESs and HDESs, respectively, contributed to sharper peaks and improved detector responses for various chiral drugs. Supporting these findings, the seminal work by Mu et al. [2] confirmed that adding a DES to a β-CD system improved the enantioresolution of zopiclone, salbutamol, and amlodipine by up to four-fold, with stronger and more defined UV detection signals.

The practical utility and broad applicability of DES-modified systems are arguably their most compelling advantages. They have been successfully employed to resolve an extensive range of analytes, encompassing structurally diverse pharmaceuticals (including β-blockers, azole antifungals, antidepressants, and stimulants), complex natural products (such as phenolic acids and flavonoids), and challenging compound classes like naphthalene derivatives, amino acids, and numerous fluorinated amphetamine and cathinone analogs. This success has been demonstrated across a wide spectrum of CE modes and conditions, utilizing capillaries with internal diameters of 25–50 µm and common BGEs such as borate, phosphate, and TRIS buffers. The remarkable adaptability of these systems is further highlighted by their effective operation across an exceptionally broad pH range, from highly acidic (pH 2.5) to strongly basic (pH 11.0), underscoring that DESs are not niche additives but robust modulators capable of enhancing separation performance under a vast array of analytical conditions. Importantly, DESs have demonstrated compatibility with a variety of capillary electrophoresis techniques, including CZE, MEKC, MEEKC, CDEKC, and NACE. Furthermore, their use is not limited by detection methods, as DES-modified systems have been effectively coupled with both UV and amperometric detectors. This versatility further reinforces the position of DESs as powerful and universal tools for advancing chiral and achiral separation science. Detailed data has been collected in Table 2 and Table 3.

### 7.2. Weaknesses of DESs

Despite the growing interest in applying DESs, their broader implementation is impeded by several physicochemical limitations. The most frequently cited barriers are the high viscosity and, in some cases, limited electrical conductivity of DES mixtures—properties that directly affect separation efficiency, analysis time, and electric field stability.

DESs, particularly those based on glycerol, sorbitol, or urea, possess markedly greater dynamic viscosities than conventional buffers; typical values can exceed 50–100 mPa·s, whereas aqueous buffers are usually below 2 mPa·s. This high viscosity is a primary cause of several adverse consequences, most notably the suppression of EOF, which prolongs analyte migration times and can reduce separation efficiency. It also reduces the electrophoretic mobility of analytes by increasing frictional drag and can introduce technical difficulties during sample injection. To mitigate these effects, DESs are typically diluted with water (10–30% *v*/*v*) to achieve a compromise between their beneficial properties and acceptable flow resistance. This EOF suppression has been consistently reported. Zhang et al. [22] attributed the slowdown to interactions between DES components and capillary wall silanol groups, while Mu et al. [2] ascribed it to a combination of surface charge neutralization and increased medium viscosity.

A second major limitation is the relatively low electrical conductivity of many DESs. Neat DESs can have conductivities in the range of 0.1–1 mS cm^−1^, markedly below the 10–30 mS cm^−1^ characteristic of standard CE buffers. This deficiency can lead to lower electrophoretic currents, unstable electric fields, and consequently, poor repeatability of migration times. As reported by Ding et al. [5], a Pro-U30 DES-based BGE generated a weaker and more unstable current compared to conventional aqueous systems. Therefore, practical applications typically employ aqueous DES mixtures supplemented with a conventional buffer salt to maintain adequate conductivity.

Finally, a frequently overlooked limitation is the occurrence of non-specific interactions between DES constituents and the inner surface of the capillary. Standard fused-silica capillaries possess a negatively charged surface due to the dissociation of silanol (-SiOH) groups. In the presence of DESs—especially those containing components like choline, lactic acid, or urea—the surface properties can be markedly altered. As Liu et al. [20] underscored, SUPRADESs can adsorb onto the capillary wall, shielding the surface charge and attenuating the EOF. When the β-cyclodextrin/L-lactic acid ratio was varied from 1:2 to 1:10, the EOF marker migration time decreased from 105 min to 95 min, indicating an increase in EOF as the acidic component grew, likely by increasing the net negative charge. In summary, DES adsorption can neutralize –SiO^−^ sites, reduce—or in some cases, reverse—the EOF, and increase its instability, thereby compromising migration reproducibility.

## 8. Conclusions

The application of DESs (including SUPRADES and HDES) in capillary electromigration techniques has rapidly evolved from a niche concept into a vibrant and versatile field of analytical chemistry. This review has demonstrated that DESs are not monolithic entities but highly tunable systems that can function in diverse roles: as chiral additives, primary separation media, sophisticated components of PSP, or PSP itself. The strategic selection of HBA/HBD components and the control of experimental conditions, such as pH, allow for the rational design of DES-based systems with anionic, cationic, or neutral characteristics, profoundly influencing both separation mechanisms and analyte migration.

While significant challenges related to viscosity and conductivity remain, the demonstrable advantages in enhancing selectivity, improving solubility, and promoting green analytical principles are undeniable. However, the application of DESs in capillary electromigration techniques, while already impactful, is still in a stage of rapid evolution. Several compelling frontiers are emerging that promise to further expand their utility and solidify their role in modern analytical chemistry.

One particularly promising research direction is the development of task-specific SUPRADES for chiral separations. Building upon recent successes, the rational design of SUPRADES as highly selective chiral phases is key. Future work will likely focus on moving beyond simple cyclodextrin–acid combinations to incorporate a wider range of chiral selectors (e.g., antibiotics, proteins, or chiral ionic liquids) as integral HBA or HBD components. The goal is to create SUPRADES tailored for a particular class of enantiomers, though a key challenge remains: the in-depth elucidation of the complex chiral recognition mechanisms within these systems.

Another highly practical future direction involves leveraging DES as a reaction medium for pre-column derivatization. Many essential derivatizing agents, such as fluorescein isothiocyanate (FITC) for amines or 8-aminopyrene-1,3,6-trisulfonic acid (APTS) for carbohydrates, exhibit limited solubility and can be prone to hydrolysis or degradation in purely aqueous media, often requiring organic solvents to proceed efficiently. By replacing these solvents with a rationally designed DES, it may be possible to create a system where the DES acts as a highly efficient reaction solvent—solubilizing both the analyte and the labeling reagent while protecting it from decomposition.

Crucially, to solidify the position of DESs as a cornerstone of modern, sustainable separation science, a transition to the analysis of real-world biological samples is necessary. To date, the vast majority of studies have used standard solutions. The next step is to validate these methods with complex biological matrices such as plasma, urine, or tissue extracts. Future studies must focus on developing and validating complete analytical workflows—from DES-assisted sample extraction to separation—proving that these green and tunable systems are not just an academic curiosity, but robust and reliable tools for routine clinical, forensic, and environmental analysis. Successfully demonstrating their ability to overcome challenges like matrix effects and protein interference will ultimately confirm the practical impact of DESs and fulfill their potential.

## Figures and Tables

**Table 1:** Compositions and stoichiometric ratio of DESs used in CE.

DES	HBA	HBD	Stechiometric Ratio (HBA:HBD)	Water/Organic Solvent Addition	Reference
ChCl-U	ChCl	U	Not stated	---	[2]
ChCl-EG		EG		---	
ChCl-PG		PG		---	
ChCl-BG		BG		---	
ChCl-U	ChCl	U	1:2	---	[3,8,9,10,11,12]
ChCl-LA		LA		---	
ChCl-EG		EG		---	
ChCl-PG		PG		---	
ChCl-G		G		---	
ChCl-CP	ChCl	CP	1:1	5 drops of water	[4]
Pro-U	Pro	U	1:3	---	[5]
Pro-U30				30% (*m*/*m*) water	
HP-β-CD-MA	HP-β-CD	MA	1:1	1 drop of MeOH	[6]
HP-β-CD-LA		LA		---	
M-β-CD-MA	M-β-CD	MA		1 drop of MeOH	
M-β-CD-LA		LA		---	
B-U	B	U	1:2:2 *	---	[7]
B-U30				30% (*m*/*m*) water	
ChCl-D-Sor	ChCl	D-Sor	1:1	---	[9,10,11,13]
ChCl-D-Glu		D-Glu	1:2	---	
ChCl-D-Fru		D-Fru	1:2	---	
TBABr-*L*-Arg	TBABr	*L*-Arg	1:1	---	[14]
TBABr-*L*-Ala		*L*-Ala		---	
TBABr-*L*-Pro		*L*-Pro		---	
(–)-Men:OA	(–)-Men	OA	1:2	---	[15,16]
S-β-CD-CA	S-β-CD	CA	1:2	---	[17,18]
N_8881_Cl:OA	N_8881_Cl	OA	1:1	---	[19]
β-CD-*L*-LA	β-CD	*L*-LA	1:3 **	---	[20]
HP-β-CD-*L*-LA	HP-β-CD	*L*-LA		---	
M-β-CD-*L*-LA	M-β-CD	*L*-LA		---	
*D*,*L*-Men:O	*D*,*L*-Men	O	1:1	---	[21]
*L*-Val-G	*L*-Val	G	1:5	---	[22]
*L*-Leu-G	*L*-Leu			---	
*L*-Val-*L*-LA	*L*-Val	*L*-LA		---	
*L*-Leu-*L*-LA	*L*-Leu			---	
TEACl-LbA	TEACl	LbA	1:2 **	---	[23]
TBACl-LbA	TBACl			---	
TBPCl-LbA	TBPCl			---	
Pro-U20	Pro	U	1:3	20% (*m*/*m*) water	[24]
ChCl-LA	ChCl	LA	1:2	---	[25]
ChCl-MA		MA		---	
ChCl-TA		TA		---	
ChCl-MA-LA		MA, LA	1:1:1	---	
ChCl-LA-TA		LA, TA		---	
ChCl-MA-TA		MA, TA		---	
MD-G	MD	G	Different weight ratios	---	[26]
TEACl-D-Xyl	TEACl	D-Xyl	1.97:1.21 ***	---	[27]
TEACl-D-Sor		D-Sor	1.97:1.22 ***	---	

*—in reaction mixture was also water; **—optimal ratio; ***—ratio calculated from corresponding masses; EG—ethylene glycol; PG—propylene glycol; BG—1,3-butylene glycol; G—glycerol; LA—lactic acid; HP-β-CD—2-hydroxypropyl-β-cyclodextrin; M-β-CD—methyl-β-cyclodextrin; MeOH—methanol; D-Sor—D-sorbitol; D-Glu—D-glucose; D-Fru—D-fructose; TBABr—tetrabutylammonium bromide; (–)-Men—menthol; OA—octanoic acid; S-β-CD—sulfated-β-cyclodextrin; CA—citric acid; N_8881_Cl—methyltrioctylammonium chloride; O—*n*-octanol; TEACl—tetraethylammonium chloride; TBACl—tetrabutylammonium chloride; *L*-Arg—*L*-arginine; *L*-Ala—*L*-alanine; TBPCl—tetrabutylphosphonium chloride; LbA—lactobionic acid; TA—tartaric acid; MD—maltodextrin; D-Xyl—D-xylitol.

**Table 2:** Overview of DESs as chiral selectors and separation enhancers in CE.

DES * Concentration in BGE	BGE	Capillary	Analytes	Technique	Detection	Reference
1.0% (*v*/*v*) ChCl-U	100 mM Tris-H_3_PO_4_, 15 mM β-CD, pH 2.5	Capillary 50.0 cm (42.0 cm effective lenght), I.D. = 50 µm	Zopiclone	DES/CD-CZE	UV	[2]
	30 mM borate-H_3_PO_4_, 7 mM β-CD, pH 4.0		Salbutamol			
	30 mM Tris-H_3_PO_4,_ 10 mM β-CD, pH 2.5		Amlodipine			
1.5% (*v*/*v*) ChCl-EG or CHCl-LA	40 mM NaH_2_PO_4_-H_3_PO_4_, 10 mM HP-β-CD, pH 2.5	Capillary 50.0 cm (41.5 cm effective lenght), I.D. = 50 µm	Tropicamide, homatropine, ofloxacin, atenolol, propranolol	DES/CD-CZE	UV	[3]
1.0% (*v*/*v*) ChCl-U or ChCl-G	30 mM NaH_2_PO_4_-H_3_PO_4_, 6.5 mM CM-β-CD, pH 4.0			DES-CDEKC		
65 mM ChCl-CP	MeOH solution containing 10% (*v*/*v*) NaOH water solution (0.3 M), 45 mM Tris	Capillary 50.0 cm (41.0 cm effective lenght), I.D. = 50 µm	Propranolol, betaxolol, clenbuterol, metoprolol, bioprolol, atenolol, bambuterol, synephrine, salbutamol, sotalol, acebutolol, procaterol	DES-NACE	UV	[4]
6.66–8.0% (*v*/*v*) HP-β-CD-MA or HP-β-CD-LA or M-β-CD-MA or M-β-CD-LA	30 mM NaH_2_PO_4_, 30% (*v*/*v*) methanol, pH 2.6	Capillary 50.0 cm (41.0 cm effective lenght), I.D. = 50 µm	Amlodipine, terbutaline, nefopam, econazole, homatropine	SUPRADES-CZE	UV	[6]
0.3% (*v*/*v*) ChCl-U	20 mM HP-β-CD, 30 mM Na_2_B_4_O_7_-H_3_PO_4_ buffer (pH 3.00)	Capillary 60.0 cm (not stated effective lenght), I.D. = 25 µm	Adrenaline, salbutamol, isoproterenol, norepinephrine, tertbutaline	DES/CD-CZE	AD	[8]
0.5% (*v*/*v*) ChCl-U	10 mM CM-β-CD, 30 mM NaH_2_PO_4_-H_3_PO_4_ buffer (pH 4.00)			DES-CDEKC		
0.1–0.5% (*m*/*v*) ChCl-D-Sor or ChCl-D-Glu or ChCl-D-Fru or ChCl-U or ChCl-EG	100 mM formate buffer (pH 3.0), 12.5 mM CM-γ-CD	Capillary 58.5 cm (50.0 cm effective length), I.D. = 50 µm	Clopidogrel	DES-CDEKC	UV	[9]
	100 mM borate buffer (pH 9.0), 5 mM SB-β-CD					
0.8–1.7% (*m*/*v*) ChCl-D-Sor or ChCl-D-Glu or ChCl-D-Fru or ChCl-U or ChCl-EG	15 mM CE-β-CD + 10 mM M-γ-CD, 100 mM borate buffer (pH 9.0)	Capillary 58.5 cm (50.0 cm effective length), I.D. = 50 µm	Dimethenamid	DES-CDEKC	UV	[10]
0.005–1.5% (*v*/*v*) ChCl-D-Sor or ChCl-D-Glu or ChCl-D-Fru or ChCl-U or ChCl-EG or ChCl-BG	20 mM NaH_2_PO_4_, 15 mM CM-β-CD, pH 2.5	Capillary 64 cm (55.5 cm effective lenght), I.D. = 50 µm	Amphetamine, methamphetamine, 3-fluorethamphetamine	DES-CDEKC	UV	[11]
	20 mM NaH_2_PO_4_, 1 mM SCF-6, pH 2.5					
0.15% (*v*/*v*) ChCl-EG	13.84 mM CM-β-CD, 20 mM NaH_2_PO_4_, pH 2.5	Capillary 64 cm (55.5 cm effective lenght), I.D. = 50 µm	6 fluorinated amphetamine analogs	DES-CDEKC	UV	[12]
0.75% (*v*/*v*) ChCl-EG	14.36 mM CM-β-CD, 20 mM NaH_2_PO_4_, pH 2.5		3 fluorinated cathinone analogs			
0.5% (*m*/*v*) ChCl-D-Sor	100 mM Borate buffer (pH 9.0), 15 mM succinyl-β-CD	Capillary 58.5 cm (50.0 cm effective length), I.D. = 50 µm	Lacosamide	DES-CDEKC	UV	[13]
10.0% (*v*/*v*) TBABr-l-Arg or TBABr-l-Ala or TBABr-l-Pro	50 mM Na_2_B_4_O_7_ (pH 9.5) 15 mM β-CD	Capillary 64 cm (55.5 cm effective lenght), I.D. = 50 µm	Methionine	DES/CD-CZE	UV	[14]
0.5% (*v*/*v*) S-β-CD-CA	20 mM phosphate buffer pH 2.5	Capillary 64 cm (55.5 cm effective lenght), I.D. = 50 µm	6 fluorinated amphetamines analogs	SUPRADES-CDEKC	UV	[17]
0.05–0.075% (*v*/*v*) S-β-CD-CA	20 mM phosphate buffer pH 2.5	Capillary 64 cm (55.5 cm effective lenght), I.D. = 50 µm	nefopam, and five cathinone derivatives	SUPRADES-CDEKC	UV	[18]
0.1% (*v*/*v*) S-β-CD-CA	100 mM Tris/10 mM Borate pH 8.0					
5.0–15.0% (*v*/*v*) β-CD-L-LA or HP-β-CD-L-LA or M-β-CD-L-LA	30 mM NaH_2_PO_4_, pH 3.0–4.0	Capillary 50.0 cm (40.0 cm effective lenght), I.D. = 50 µm	Carvedilol, miconazole, clenbuterol, tertbutaline, iconazole, econazole, tioconazole, chlorpheniramine, brompheniramine, propranolol	SUPRADES-CZE	UV	[20]
0.5% (*m*/*v*) L-Val-G or L-Leu-G or L-Val-L-LA or L-Leu-L-LA	50 mM Tris/H_3_ PO_4_, 4.0% maltodextrin, pH 3.0	Capillary 50.0 cm (40.5 cm effective length), I.D. = 50 µm	Nefopam, ketoconazole, citalopram, doxapram,	DES/CS-CZE	UV	[22]
120 mM TBACl-LbA	40 mM borax buffer, 30% MeOH, pH 8.0	Capillary 50.0 cm (41.0 cm effective length), I.D. = 50 µm	20 model drugs (hydrochloride salts)	DES-CZE	UV	[23]
0.3–0.6% (*m*/*v*) ChCl-LA or ChCl-MA or ChCl-TA or ChCl-MA-LA or ChCl-LA-TA or ChCl-MA-TA	40 mM borax buffer, 20% methanol (*v*/*v*), pH 8.0	Capillary 50.0 cm (41.5 cm effective length), I.D. = 50 µm	Carbinoxamine, nefopam, propranolol, citalopram	DES/CS-CZE	UV	[25]
4.0–8.0% (*m*/*v*) MD-G	50 mM Tris/H_3_PO_4_ buffer (50 mM Tris), pH 3.0	Capillary 50.0 cm (41.0 cm effective length), I.D. = 50 µm	6 azole antifungal drugs	DES-CZE	UV	[26]
0.5% (*m*/*v*) CNTs modified using TEACl-D-Xyl or TEACl-D-Sor	50 mM Tris/H_3_PO_4_ buffer (pH 3.0), 3.0% (*m*/*v*) MD	Capillary 50.0 cm (I.D. = 50 µm)	Doxapram, duloxetine, citalopram, fluoxetine	DES/CNT-CZE	UV	[27]

*—for type of DES see Table 1; I.D.—internal diameter; CZE—capillary zone electrophoresis; SB-β-CD—sulfobutylated β-CD; CM-γ-CD—carboxymethyl-γ-CD; CS—chiral selector.

**Table 3:** Applications of DESs as separation media in capillary electrophoresis.

Type of DES	BGE	Capillary	Analytes	Technique	Detection	Reference
Pro-U30	50 mM TRIS in Pro-U30	Capillary 50.0 cm (41.0 cm effective lenght), I.D. = 50 µm	Naphthalene, 1-(naphthalen-1-yl)-ethanamine, 8 naphtoic acids	DES based CZE	UV	[5]
Pro-U30	50 mM TRIS in Pro-U30, 2% Tween-20			DES based MEKC		
B-U30	50 mM TRIS in BU30, pH 9.5	Capillary 50.0 cm (41.0 cm effective lenght), I.D. = 50 µm	10 naphthalene-based compounds	DES based CZE	UV	[7]
	50 mM TRIS in BU30, 0.3–0.5% SDS, pH 10.0			DES based MEKC		
0.2% (*m*/*v*) Men:OA	10 mM borax, (pH 9.5)	Capillary 50.0 cm (41.5 cm effective lenght), I.D. = 50 µm	13 naphthalene-based acids	HDES-EKC	UV	[15]
0.3% (*m*/*v*) (–)-Men:OA	10 mM borax, (pH 9.24), 5% HP-γ-CD			CD-HDES-EKC		
0.3% (*m*/*v*) (–)-Men:OA	20 mM phosphate buffer (pH 2.5), 2.0–4.0% CM-β-CD	Capillary 50.0 cm (41.5 cm effective lenght), I.D. = 50 µm	Citalopram, nefopam, tioconazole, isoconazole, miconazole, econazol	CD/HDES-EKC	UV	[16]
0.20% (*v*/*v*) N_8881_Cl:OA	20 mM phosphate buffer (pH 2.5),1% CM-β-CD *	Capillary 50.0 cm (41.5 cm effective lenght), I.D. = 50 µm	Tioconazole, Isoconazole, miconazole, econazole	CD/HDES-EKC	UV	[19]
0.5% D,L-Men:O	3.3% SDS, 6.6% 1-butanol, 10 mM borax buffer	Capillary 55.0 cm (46.5 cm effective lenght), I.D. = 50 µm	Luteolin, rutin, quercetin, hyperoside, chlorgenic acid, ferulic acid, isoquercitrin, caffeic acid, gallic acid	DES/W MEEKC	UV	[21]
Pro-U30	1.5–10.0% (*m*/*v*) β-CD, 50 mM H_3_BO_3_ in Pro-U30, (apparent) pH 8.0	Capillary 50.0 cm (41.0 cm effective lenght), I.D. = 50 µm	8 naphtoic acids	DES based CZE	UV	[24]

*—most effective system.

## Data Availability

Data are contained within the article.
